# Genome-wide identification and expression analysis of the BAHD gene family in *Leonurus japonicus*

**DOI:** 10.3389/fgene.2024.1512692

**Published:** 2024-12-19

**Authors:** Qing Wang, Tongtong Guo, Yuxiao Yi, Jiaxin Zhang, Wenhan Lv, Fengtang Yang, Jianing Xu

**Affiliations:** School of Life Sciences and Medicine, Shandong University of Technology, Zibo, Shandong, China

**Keywords:** acylation, BAHD acyltransferases, *L. japonicus*, secondary metabolites, gene family

## Abstract

Acylation represents a pivotal biochemical process that is instrumental in the modification of secondary metabolites throughout the growth and developmental stages of plants. The BAHD acyltransferase family within the plant kingdom predominantly utilizes coenzyme A thioester as the acyl donor, while employing alcohol or amine compounds as the acceptor substrates to facilitate acylation reactions. Using bioinformatics approaches, the *LjBAHD* gene family members in the genome of *Leonurus japonicus* (*L. japonicus*) were identified and characterized including gene structure, conserved motifs, *cis*-acting elements, and potential gene functions. To elucidate the roles of BAHD genes in various tissues of *L. japonicus*, the expression profiles of *LjBAHD* family members across different organs were scrutinized. Under drought stress treatment, some *LjBAHDs* were upregulation, suggesting their potential involvement in drought response. Notably, a detailed study was conducted on a specific HCT gene (i.e., *LjBAHD*25) within the BAHD gene family. Analysis of its expression patterns suggested a role for *LjBAHD*25 in the phenylpropanoid metabolism pathway in *L. japonicus*, contributing to the biosynthesis of secondary metabolites with unique bioactivity. The findings of this study have established a scientific foundation for the subsequent development and functional validation of the BAHD gene family in *L. japonicus.*

## 1 Introduction

Secondary metabolites endowed with specialized bioactivity are integral to the growth and developmental processes in plants. These metabolites typically undergo a series of modifications to manifest their unique effects. Acylation stands out as a critical step in this modification process (Moglia et al., 2016), with the BAHD acyltransferase family being instrumental in the biosynthesis of these compounds. The BAHD family, one of the most extensive metabolic protein families in terrestrial plants, derives its name from the first letters of four signature enzymes: benzyl alcohol O-acetyl transferase (BEAT), anthocyanin O-hydroxycinnamoyl transferases (AHCTs), anthranilate N-hydroxycinnamoyl/benzoyltransferase (HCBT), and deacetylvindoline 4-O-acetyl transferase (DAT) (St-Pierre and Luca, 2000). The BAHD family is characterized by two highly conserved motifs: HXXD and DFGWG (Liang et al., 2022). The HXXD motif is pivotal in the catalytic mechanism and is recognized as a key active site. Interestingly, the DFGWG motif is situated away from the active center and, while seemingly serving a structural function, is essential for the enzyme’s proper activity (El-Sharkawy et al., 2005). It is noteworthy that, despite the low sequence similarity in their primary structures (approximately 15%–30% identity), BAHD acyltransferases exhibit a degree of similarity in their tertiary structures.

It is well-documented that BAHD is involved in the biosynthesis of a spectrum of natural secondary metabolites, including flavonoids, phenols, alkaloids, and anthocyanins, is well-documented (D’Auria, 2006). For instance, Bernard *et al.* demonstrate that the co-expression of two BAHD family members, *CiSHT1* and *CiSHT2*, promotes tetracoumaroyl spermine accumulation in chicory (Bernard et al., 2022). Additionally, BAHD has been shown to play a important role in the synthesis of phenolamides. Peng et al. illustrated that variations in the BAHD N-acyltransferases in rice contribute to the natural diversity of aromatic amine conjugates (Peng et al., 2016). Moreover, there is a growing body of evidence indicating that acylation modifications are also crucial for plant growth, reproduction, and adaptation to biotic and abiotic stresses (Moghe et al., 2023).

Hydroxycinnamoyl transferase (HCT) constitutes a notable branch within the plant acyltransferase family and was initially isolated and characterized from tobacco. Hoffmann’s pioneering work identified dual enzymatic activities associated with this protein, pertaining to shikimic acid hydroxycinnamoyl transferase and quinic acid hydroxycinnamoyl transferase (Hoffmann et al., 2004), leading to its eponymous classification. Subsequent research on HCT has elucidated its capacity to utilize an array of acyl-CoA substrates, including cinnamoyl-CoA, p-coumaroyl-CoA, caffeoyl-CoA, feruloyl-CoA, and mustard-acyl-CoA, as acyl donors. These reactions catalyze a diverse set of acceptor molecules such as shikimic acid, quinic acid, 4-hydroxyphenylacetic acid, gentisic acid, and 4-hydroxyphenylacetamine, culminating in the formation of ester or amide compounds (Molina and Kosma, 2014; Werner and Petersen, 2019).

HCT has been implicated in the biosynthesis of lignin, a critical structural polymer in the plant cell wall (Dong et al., 2022), and chlorogenic acid (Zhao et al., 2019), an active compound with notable biological properties. Furthermore, the role of HCT extends to defense mechanisms against pathogen invasion in plant, highlighting its multifaceted importance in plant physiology (Zhang et al., 2018). The acylated products generated by HCT are known to enhance the physicochemical characteristics and biological activities of plant secondary metabolites. Consequently, HCT enzymes are extensively utilized in the development of biomass energy, the enhancement of crop varieties, and the formulation of anti-inflammatory pharmaceuticals. HCT plays a crucial role in the synthesis and post-modification of plant secondary metabolites, and it holds significant potential for future research and application (Qin et al., 2019).

*Leonurus japonicus* (*L. japonicus*), a member of the Labiatae family, is a perennial herbaceous flowering plants, extensive geographical distribution across the globe (Bernard G. et al., 2022). Traditional Chinese medicine has long recognized the aerial parts of *L. japonicus* as a valuable medicinal resource, particularly for the treatment of various gynecological conditions. These include dysmenorrhea, amenorrhea, menstrual irregularities, and *postpartum* hemorrhage, with documented applications dating back over 1800 years (Miao et al., 2019). With modern pharmacological research continuing to explore the therapeutic potential of *L. japonicus*, it has become evident that this plant possesses a range of beneficial properties, extending beyond gynecological applications to positive effects on the cardiovascular, reproductive, and nervous systems (Liu et al., 2017). The secondary metabolites in *L. japonicus*, which include alkaloids, diterpenoids, and flavonoids, have been shown to exert important bioactivity. They contribute to antioxidation, anti-inflammatory, and cardioprotective actions, as well as provide protection against myocardial damage (Shang et al., 2014; Wang et al., 2017). The multifaceted medicinal uses of *L. japonicus* underscore the importance of continued research into its bioactive constituents and their potential applications in the development of novel therapeutics.

Genome-wide analyses offer profound insights into the genetic underpinnings of secondary metabolite synthesis in plants. To date, the genomes of the BAHD acyltransferase family have been delineated in a variety of species, including the *Welsh Onion* (Liu et al., 2023), *Wurfbainia villosa* (Liang et al., 2022), *Lithospermum erythrorhizon* (Wang et al., 2022), and *Theobroma cacao* (Abdullah et al., 2021a). Despite these advances, research on the BAHD gene family within *L. japonicus* remains scarce. The complete genome sequence for *L. japonicus* marks a significant milestone in molecular biology research. This genomic resource is instrumental for advancing our understanding of the biosynthetic pathways that produce the plant’s bioactive medicinal constituents. It also facilitates the exploration of strategies to enhance the medicinal value of *L. japonicus*. With the genome sequence in hand, researchers are now better equipped to identify and characterize the BAHD gene family members, which are likely to play a pivotal role in the synthesis of the plant’s therapeutically relevant secondary metabolites.

In the present investigation, to identify *LjBAHD* genes in the *L. japonicus* genome, a comprehensive bioinformatic analysis was conducted, including a detailed examination of the genes structures, the identification of conserved motifs, the analysis of *cis*-acting regulatory elements, and the elucidation of gene functions. By scrutinizing the characteristics of the *LjBAHD* genes and correlating these with their distinct expression profiles across various organs of *L. japonicus*, we have gained valuable insights into the functional roles of BAHD genes in *L. japonicus*. A particular focus of this research was the investigation of a HCT gene, *LjBAHD*25, in *L. japonicus*. Through an in-depth analysis of the gene expression pattern of *LjBAHD*25, it was determined that this gene is actively involved in the phenylpropanoid metabolic pathway. This involvement is crucial since it provides essential precursors for the synthesis of a diverse array of secondary metabolites with specialized bioactive properties in *L. japonicus*. Thus, the findings of this study lay the groundwork for future research aimed at harnessing the potential medicinal value of *L. japonicus* and its secondary metabolites.

## 2 Materials and methods

### 2.1 Plant materials and treatment

The *L. japonicus* used in this study was a biennial variety, collected in the field in Zibo, Shandong Province, China (118.07 E,36.08 N). It was collected without any treatment during the normal growing season, under conditions free from mechanical damage and the presence of pests, and a sampling permit was obtained prior to the collection of experimental material. Immediately following collection, the samples were immersed in liquid nitrogen, transported to the laboratory, and stored in an ultra-low temperature refrigerator at −80°C for subsequent RNA-seq testing of the leaves, stems, roots, and floral organs. In the experimental setup, drought stress was induced using 40% (w/v) polyethylene glycol 6000 (PEG 6000) to simulate water deficit conditions. Leaves were chosen for transcriptomic sequencing analysis. The plant samples were kept in the laboratory of the School of Life Sciences and Medicine, Shandong University of Technology.

### 2.2 Identification and characterization of *LjBAHD* superfamily

The hidden Markov model file (PF02458) of BAHD gene family was downloaded from Pfam database (https://www.ebi.ac.uk/interpro/entry/pfam/PF03106/). Hmmer 3.0 (http://hmmer.janelia.org/) was used to screen candidate *LjBAHD* genes with e value less than E^−10^ in *L. japonicus* genome (Wang et al., 2022). In addition, SMART [SMART: Main page (embl.de)] was used to determine whether all the candidate *LjBAHDs* contain the whole BAHD domain, removed the abnormal sequence and the sequence lacking one or two characteristic motifs: HXXD and DFGWG (Liang et al., 2022). Expasy (https://web.expasy.org/compute_pi/) was used to calculate the molecular weight (MW) and isoelectric point (pI). Wolfpsort (https://wolfpsort.hgc.jp) was used to predict subcellular localization, and MEME (meme-suite.org) was used to analyze 47 conservative motifs of full-length amino acid sequences of *LjBAHDs*, and 10 motifs were set. Multiple sequence alignment was performed with DNAMAN. The TBtools v2.012 software was used to analyse and visualise the exon-intron structure and chromosomal localization of *LjBAHDs*.

### 2.3 Phylogenetic tree construction

The sequence of *LjBAHDs* of *L. japonicus* and *Arabidopsis thaliana* was compared by the method of muscle, and the BAHDs of *A. thaliana* were from TAIR (arabidopsis.org). Maximum Likelihood (ML) tree with 1,000 bootstrap replications was constructed with MEGA 11.0 software with WAG + G + I + F model. Subsequently, the constructed phylogenetic tree was modified with ITOL (http://itol.embl.de). BAHD classification related classification is based on the previously reported classification of *A. thaliana* BAHD gene family (Liang et al., 2022).

### 2.4 Analysis of intra-species collinearity and inter-species collinearity

Using MCScanX to check gene replication events with default parameters (Liu et al., 2023). TBtools was used to analyze the inter-species collinearity of BAHD genes of *L. japonicus*, *A. thaliana* and *Salvia bowleyana*, and advanced Circos of TBtools was used to analyze the intra-species collinearity of *L. japonicus*. The synonymous mutation rate (Ks), non-synonymous mutation rate (Ka) and the ratio of non-synonymous mutation rate to synonymous mutation rate (Ka/Ks) of *LjBAHDs* repeat gene in *L. japonicus* were calculated by using kaks _ calculator tool.

### 2.5 Promoter *cis*-acting element and annotation analysis of *LjBAHDs*

The *cis*-acting elements of the 2000 bp promoter sequence of *LjBAHDs* were analyzed using the PlantCARE database (http://bioinformatics.psb.ugent.be/webtools/plantcare/html/), The Cytoscape v3.7.1 was used to draw the relationship between the *LjBAHDs* and the transcription factors, the transcription factors were dived into three types represented by three colors: related to growth and development (green), abiotic stress (red) and related to growth and stress (cyan).

### 2.6 GO and KEGG annotation analysis of *LjBAHDs*

GO and KEGG enrichment analysis were performed for *LjBAHDs* genes to further analyse their biological functions, through a pipeline on the Omicshare Bioinformatics Cloud Platform (GENEDENOVO, Guangzhou, China).

### 2.7 Gene expression analysis of *LjBAHDs*

To explore the specific expression patterns of *LjBAHDs* in different *L. japonicus* organs, RNA-seq data from *L. japonicu*s mature roots, leaves,stems and flowers were from our lab and used for gene expression analysis. The heatmap was drawn in Rstudio using pheatmap.

### 2.8 RNA isolation, and qRT-PCR validation

The primers were done using Primer-BLAST [Primer designing tool (nih.gov)]; (Supplementary Table S3); their synthesis was entrusted to GENEWIZ (Suzhou, China), and rRNA served as a reference gene. Total RNA was extracted from the samples using a Plant RNA Rapid Extract Kit (coolaber, Beijing, China), and cDNA was obtained using a TransScript^®^ One-Step gDNA Removal and cDNA Synthesis SuperMix (Trans Gene). The qRT-PCR was performed on a StepOne Real-Time PCR System (Applied Biosystems) using SYBR Green realtime PCR master mix (SYBR Green, Toyobo). The PCR reaction program consisted of 95°C for 30 s, followed by 40 cycles of 95°C for 10 s, 60°C for 30 s, 95°C for 15 s, and 60°C for 1 min, finishing with 95°C for 15 s. The experiment was repeated three times, the relative expression level was calculated by the 2^−ΔΔCT^ method.

### 2.9 Statistical analysis

All data are presented as means with standard deviations (SDs). Statistical analysis and bar graphs were created by GraphPad Prism 8.3.0 software. *p < 0.05, **p < 0.01.

## 3 Results

### 3.1 Identification and physicochemical analysis of BAHD gene family

**FIGURE 1:**
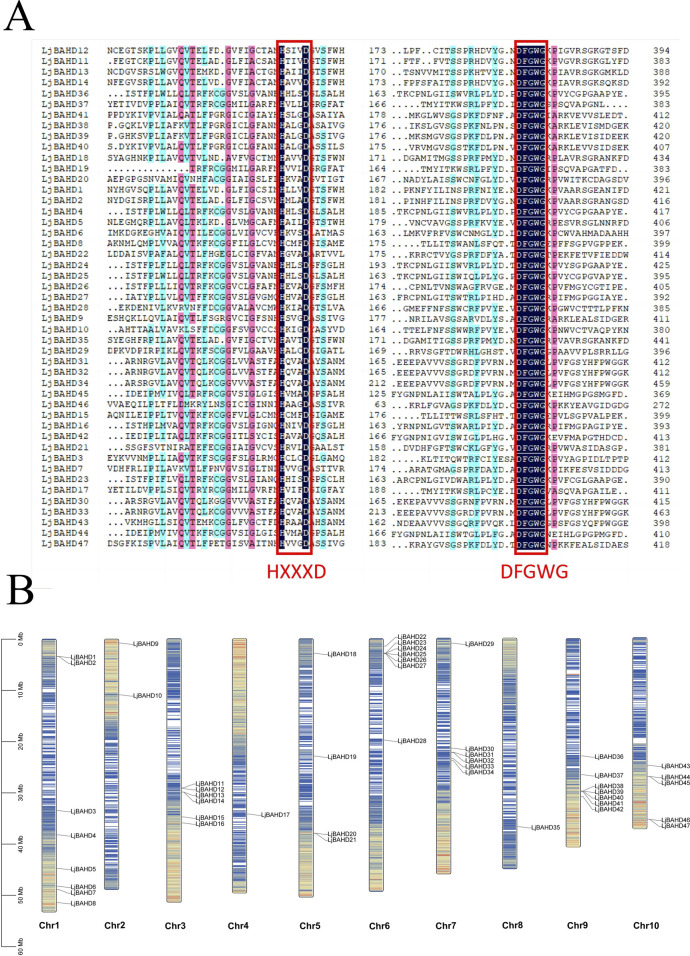
Sequence comparison and chromosomal localisation of 47 *LjBAHDs*. **(A)** Multiple sequence alignments of conserved domains in *LjBAHDs*. Red box represent the conserved BAHD domains. **(B)** Chromosome distributions of *LjBAHDs.*

To systematically identify the BAHD acyltransferase gene family in *L. japonicus*, a bioinformatics approach was employed using the Hidden Markov Model (HMM) profile corresponding to the BAHD domain (PF02458). This search against the whole genome of *L. japonicus* initially yielded 87 candidate genes. Subsequent domain validation using the SMART web tool and HMM-based filtering led to the identification of 47 *LjBAHD* genes, with each featuring intact BAHD domains and the conserved HXXD and DFGWG motifs (Figure 1A). These genes were renamed *LjBAHD1* to *LjBAHD47* according to their chromosomal location (Figure 1B). Chromosomal distribution analysis revealed an uneven distribution of *LjBAHD* genes across the genome, with a higher frequency observed on chromosomes 1, 3, 6, 7, and 9 (8, 6, 6, 6, and 7 genes respectively), and a lower frequency on 2, 4, and 8 (2, 1, and 1 genes respectively). Additionally, clustering of certain *LjBAHD* genes was observed, suggesting a potential functional relatedness, shared regulatory mechanisms, and a collaborate role in the biosynthesis of specific metabolites in *L. japonicus* (Chakraborty, 2022).

A detailed physicochemical analysis of the 47 deduced LjBAHD proteins is presented in Supplementary Table S1. This analysis highlighted a considerable variation in the number of amino acids, ranging from 311 in LjBAHD46 to 874 in LjBAHD38. Correspondingly, the relative molecular weights varied from approximately 33.8 kDa for LjBAHD46 to 96.6 kDa for LjBAHD38, with an overall average of 52.75 kDa. The theoretical isoelectric points (pI) spanned a range from 5.14 for LjBAHD46 to 8.41 for LjBAHD18. Subcellular localization predictions indicated that 46 of the LjBAHD proteins were predominantly cytoplasmic, while LjBAHD33 was predicted to be localized to the cell membrane. This distribution is in concordance with the findings of D’Auria (2006), who reported the absence of signal peptides in nearly all BAHD family members, consistent with a cytoplasmic localization. In summary, the sequence diversity and the physicochemical properties of the BAHD acyltransferase family in *L. japonicus* underscore its potential for diverse biological functions and highlight the complexity of the regulatory mechanisms underlying the synthesis of secondary metabolites in this species.

### 3.2 Phylogenetic analysis, and motif composition of the BAHD in *L. japonicus*

Exon and intron analysis is pivotal for elucidating gene structures, organizational patterns, and the functional implications of proteins. This structural information is also instrumental to phylogenetic studies, which can shed light on the evolutionary dynamics of gene families, including the gains, losses, and modifications of gene structures (Wang et al., 2013). In this context, the functional analysis of the 47 *LjBAHD* genes was extended by examining their gene structures, identifying characteristic motifs, and constructing a phylogenetic tree. The exon counts within the BAHD family genes of *L. japonicus* vary from one to four, with 15 genes being intronless and possessing a single exon (Supplementary Figure S1).

**FIGURE 2:**
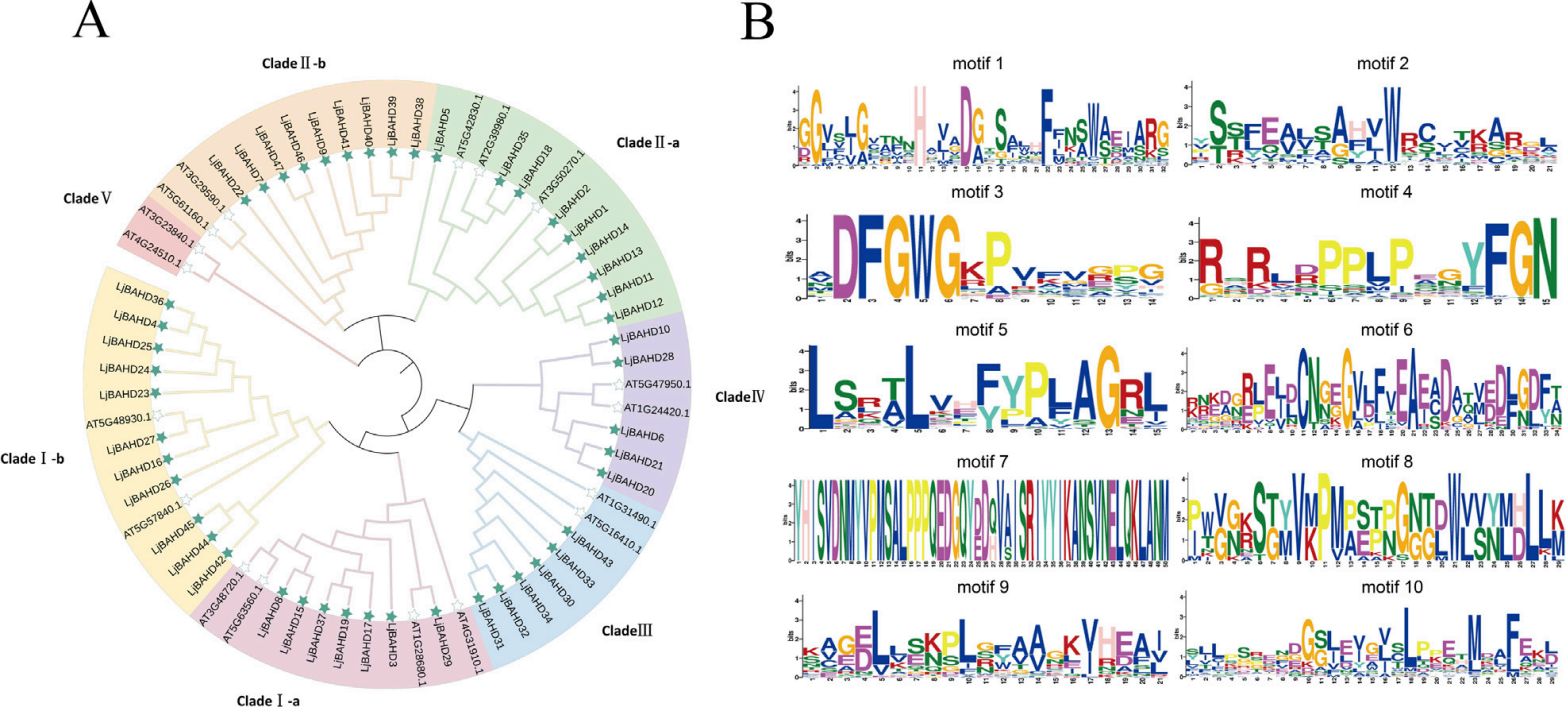
Phylogenetic tree and conserved motif of *L. japonicus*. **(A)** Phylogenetic analysis of *L. japonicus* and *Arabidopsis thaliana* BAHD domain. *L. japonicus* and *Arabidopsis thaliana* are marked as green and white pentagrams respectively. **(B)** Analysis of the conserved motifs of 47 *LjBAHDs*.

To delineate the evolutionary relationships and potential functions of the *LjBAHDs*, a phylogenetic tree (Figure 2A) was constructed using the maximum likelihood method based on 64 amino acid sequences of BAHD, encompassing 47 typical sequences from *LjBAHDs* and 17 from *Arabidopsis thaliana* (*AtBAHDs*). Following the classification by D'Auria *et al.*, BAHD family members are categorized into five distinct clades (D’Auria, 2006). Notably, the BAHD members in *L. japonicus* were classified into four clades, with a notable absence of Clade V. Clade I in *L. japonicus* comprises 18 members, with Clade I-a and Clade I-b consisting of respectively 7 and 11 members, predominantly involved in the modification of phenolic glucosides (Taguchi et al., 2005). Clade II includes 18 members, with Clade II-a and II-b having 9 members each, whose enzymes are primarily engaged in the elongation of long-chain epidermal waxes, which are crucial for reducing water loss and enhancing plant resistance to pathogens (D’Auria, 2006). Clade III consists of six members capable of accepting a diverse array of alcohol substrates (St-Pierre et al., 2002). Clade IV encompasses five members, predominantly characterized as agmatine coumaroyl transferases (D’Auria, 2006).

A total of ten conserved motifs (Motifs 1–10) within the BAHD proteins were identified using the MEME web tool (Figure 2B). These BAHD genes are marked by the conserved Motif 1 (HXXD) and Motif 3 (DFGWG). The presence of the characteristic -YFGNC- motif in BAHD members is often correlated with their roles in the biosynthesis of anthocyanins or flavonoids (Nakayama et al., 2003). Upon enumeration of the -YFGNC- motifs among the 47 *LjBAHDs*, it was determined that nine proteins contain this motif (Supplementary Table S1).

### 3.3 BAHD genes duplication and synteny analyses

**FIGURE 3:**
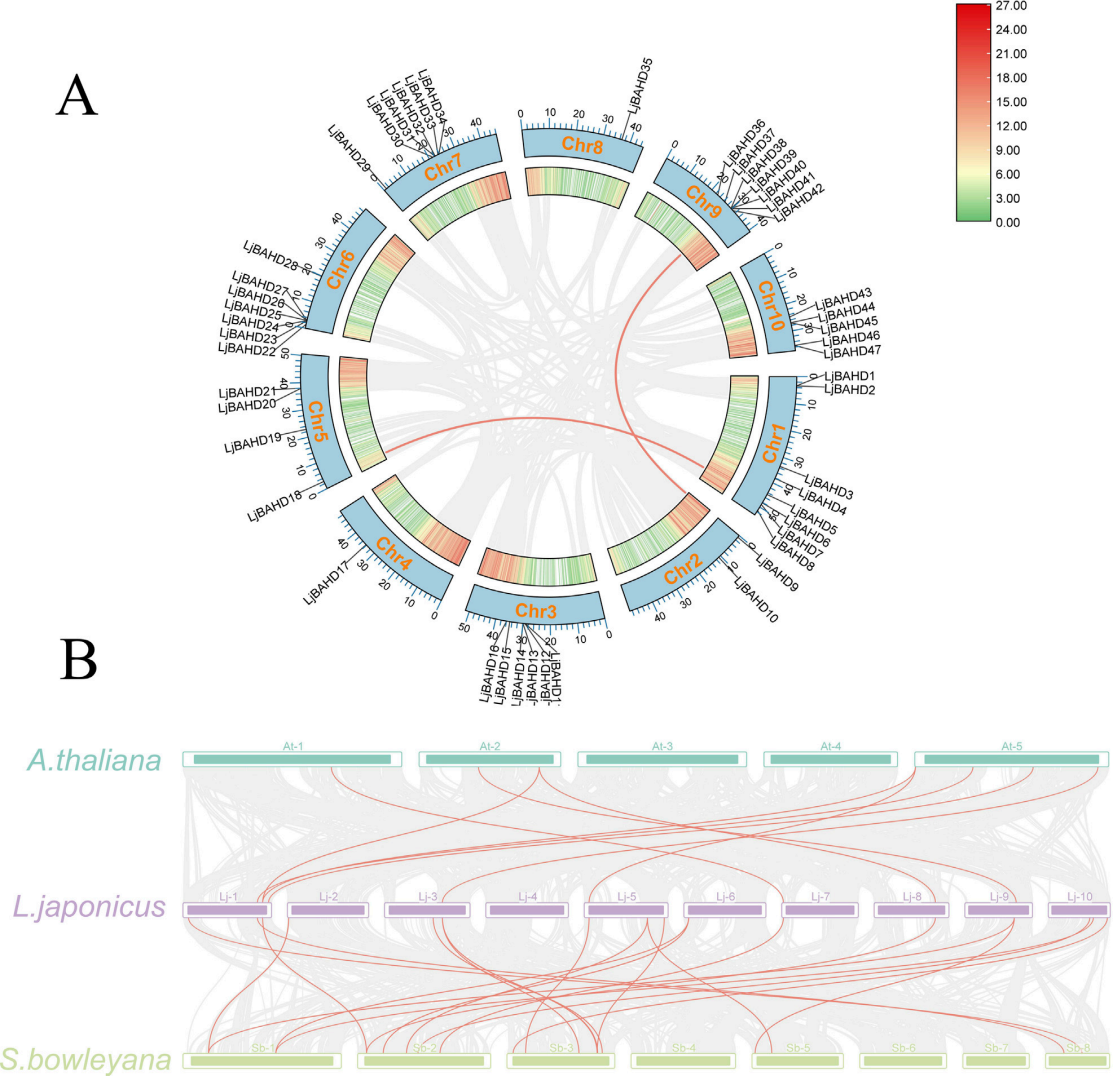
Synteny analyses. **(A)** Synteny analysis of BAHDs in *L. japonicus* genome. **(B)** Synteny analysis of BAHDs in *L. japonicus* with, *Arabidopsis thaliana*, *Salvia bowleyana*.

Duplicated genes have similar gene structures and biological functions, provide critical insights into the evolutionary and expansionary processes of gene families (Uno et al., 2000). The MCScanX algorithm was used to investigate gene duplication events within the BAHD gene family in *L. japonicus*. Syntenic regions in two chromosomal parts represented by red lines, it was observed between *LjBAHD11* and *LjBAHD18*, *LjBAHD9* and *LjBAHD38*. Considering these gene pairs residing on distinct chromosomes, it may indicate they are products of fragmental duplication events (Figure 3A). Notably, *LjBAHD6* and *LjBAHD18* were classified within Clade II-a, while *LjBAHD9* and *LjBAHD38* fall under Clade II-b in the phylogenetic tree. Since the functions of these genes are implicated in aiding environmental adaptation and survival enhancement, it may suggest that *L. japonicus* has encountered adverse environmental conditions at certain stages of its evolutionary history. To assess the selective pressures on these duplication *LjBAHD* genes, calculations of the non-synonymous substitution rate (Ka), synonymous substitution rate (Ks), and the Ka/Ks ratio were performed. The Ka/Ks ratios for fragment duplicates arising from tandem duplications were determined to be 0.1718 and 0.1727 (Supplementary Table S2). Furthermore, all co-dominant *LjBAHD* gene pairs exhibited Ka/Ks ratios less than 1, indicative of purifying selection acting upon these gene pairs.

To elucidate the evolutionary relationships among BAHD family members across different plant species, collinearity analysis was conducted between *L. japonicus* and two other species: *Arabidopsis thaliana* (*A. thaliana*) and a fellow member of the Labiatae family, *Salvia bowleyana* (*S. bowleyana*) (Figure 3B). The analysis revealed the existence of nine homologous BAHD genes between *L. japonicus* and *A. thaliana*, with a preponderance localization on chromosome 1 of *L. japonicus*. In contrast, non-homologous BAHD genes were identified on chromosomes 2, 4, 6, and 10. Moreover, twenty homologous BAHD genes were identified between *L. japonicus* and *S. bowleyana*, with a higher concentration on chromosome 5 of *L. japonicus*, and an absence on chromosome 4. These findings provide a comprehensive view of the evolutionary dynamics within the BAHD gene family of *L. japonicus*, highlighting the complex interplay between gene duplication, selective pressures, and the adaptive responses of this species to its environment.

### 3.4 Analysis of *cis*-acting elements in the promoters of *LjBAHD* genes

**FIGURE 4:**
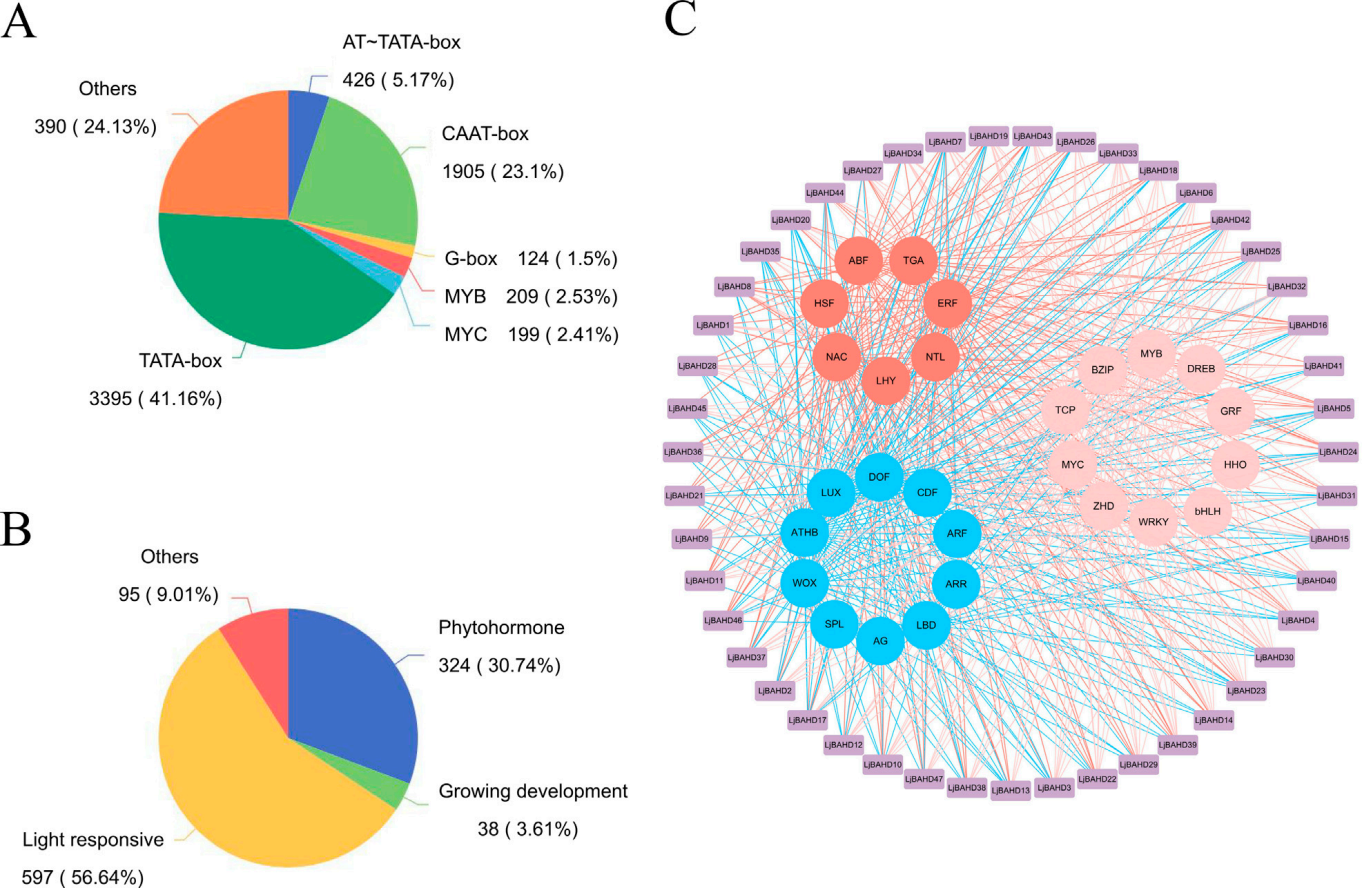
Analysis of the *cis*-acting elements of the *LjBAHDs.* **(A)** Promoter motif type statistics. **(B)** Classification of identified regulatory elements according to function and their response to hormones, light and growth. **(C)** The network of transcription factors regulating the expression of *LjBAHDs*.

To enhance our understanding of the functions and expression patterns of the *LjBAHD* gene family in *L. japonicus*, an in-depth analysis of the *cis*-acting elements within the promoter regions of the 47 *LjBAHD* genes was conducted using the PlantCARE database. The predictive analysis identified a total of 6,414 *cis*-acting elements across these promoter regions, which were predominantly categorized into eight distinct types (Figure 4A). Notably, TATA-box and CAAT-box elements were found to be the most prevalent, constituting 41.16% and 23.10% of the total elements identified, respectively.

The regulatory functions of these *cis*-acting elements allow them to be classified into four main groups: light responsiveness, plant hormone responsiveness, stress responsiveness, and plant growth and development responsiveness (Figure 4B). Visualization of the *cis*-acting elements within the promoters (Supplementary Figure S2) revealed that all *LjBAHD* promoter regions are enriched with multiple light-responsive elements. This was followed by a significant presence of hormone-responsive elements, including those responsive to jasmonic acid (CGTCA and TGACG motifs), gibberellin (CCTTTTG and TATCCCA motifs), and abscisic acid (ABRE and AAGAA motifs). Additionally, stress-stimulating elements were identified in the *LjBAHD* promoters, such as the low-temperature-responsive element (LTR), stress-responsive element (STRE), and drought-responsive element (MSB). Plant development-related elements and transcription factor binding sites for MYB and MYC were also observed.

To further investigate the transcription factors that may regulate *LjBAHD* gene expression, predictions were made based on the identified *cis*-acting elements within the promoter regions. A regulatory network of transcription factors implicated in the modulation of *LjBAHD* expression was constructed (Figure 4C). The analysis revealed that the 47 *LjBAHD* genes are potentially regulated by 27 distinct transcription factors. These transcription factors could be categorized based on their functions into three groups: growth-related transcription factors (depicted in blue), abiotic stress-related transcription factors (in red), and those with dual functions in growth and stress responses (in pink). The regulatory relationships within this network suggest that the BAHD genes in *L. japonicus* are predominantly under the control of growth-related transcription factors.

### 3.5 Functional annotation of *LjBAHD* genes

**FIGURE 5:**
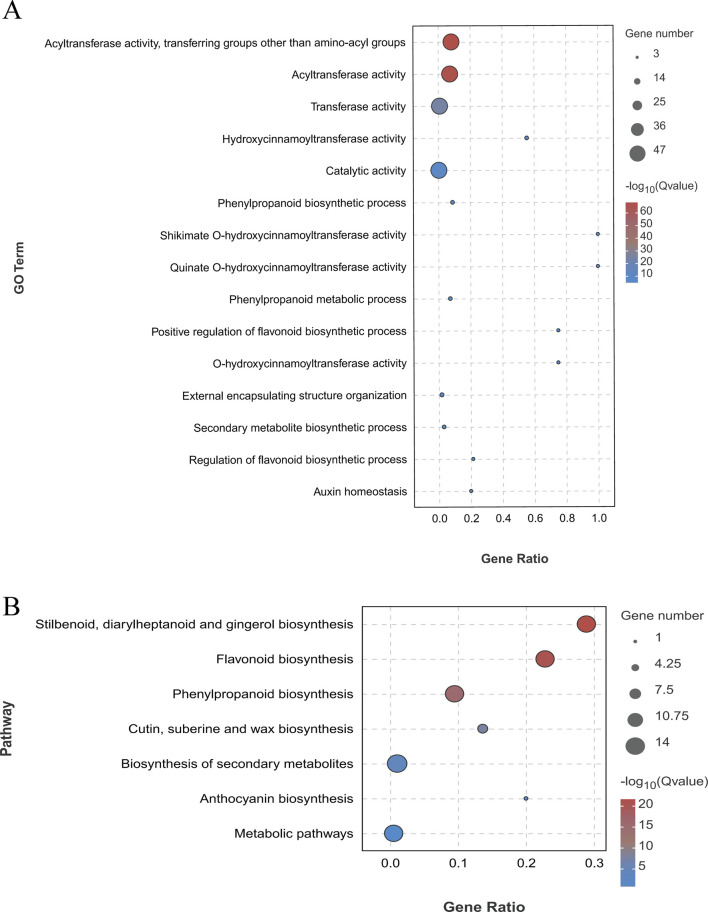
Functional annotation of *LjBAHD* genes. **(A)** Gene ontology (GO) analysis of *LjBAHDs*. **(B)** Kyoto Encyclopedia of Genes and Genomes (KEGG) analysis of *LjBAHDs.*

Gene Ontology (GO) analysis was employed to investigate the functional enrichment among the 47 *LjBAHD* genes in *L. japonicus*. The results of this analysis categorized the *LjBAHD* genes into three primary GO domains: biological processes, cellular components, and molecular functions (Supplementary Figure S3). Within the biological processes domain, a majority of *LjBAHD* genes were implicated in metabolic process (GO:0008152) and cellular processes (GO:0009987). In terms of cellular components, the *LjBAHD* genes were predominantly associated with the cellular anatomical entity (GO:0110165). When considering molecular functions, the *LjBAHD* genes were mainly enriched in catalytic activity (GO:0003824). A detailed examination of molecular function items revealed significant enrichment in acyl transfer activity, specifically the transfer of groups other than amino-acyl groups (GO:0016747), and transfer activity (GO:0016740) (Figure 5A).

Furthermore, enrichment analysis using the Kyoto Encyclopedia of Genes and Genomes (KEGG) pathway database (Figure 5B) demonstrated that the *LjBAHD* genes are significantly enriched in several key biosynthetic pathways. These include the Stilbenoids, diarylheptanoids and gingerol biosynthesis (Ko00945), Flavonoid biosynthesis (Ko00941), and Phenylpropanoid biosynthesis (Ko00940). The GO and KEGG enrichment analyses collectively provide a comprehensive overview of the potential roles and metabolic pathways associated with the *LjBAHD* genes in *L. japonicus*, underscoring their involvement in critical biological processes and cellular functions.

### 3.6 *LjBAHDs* expression analysis in different organs and drought stress

**FIGURE 6:**
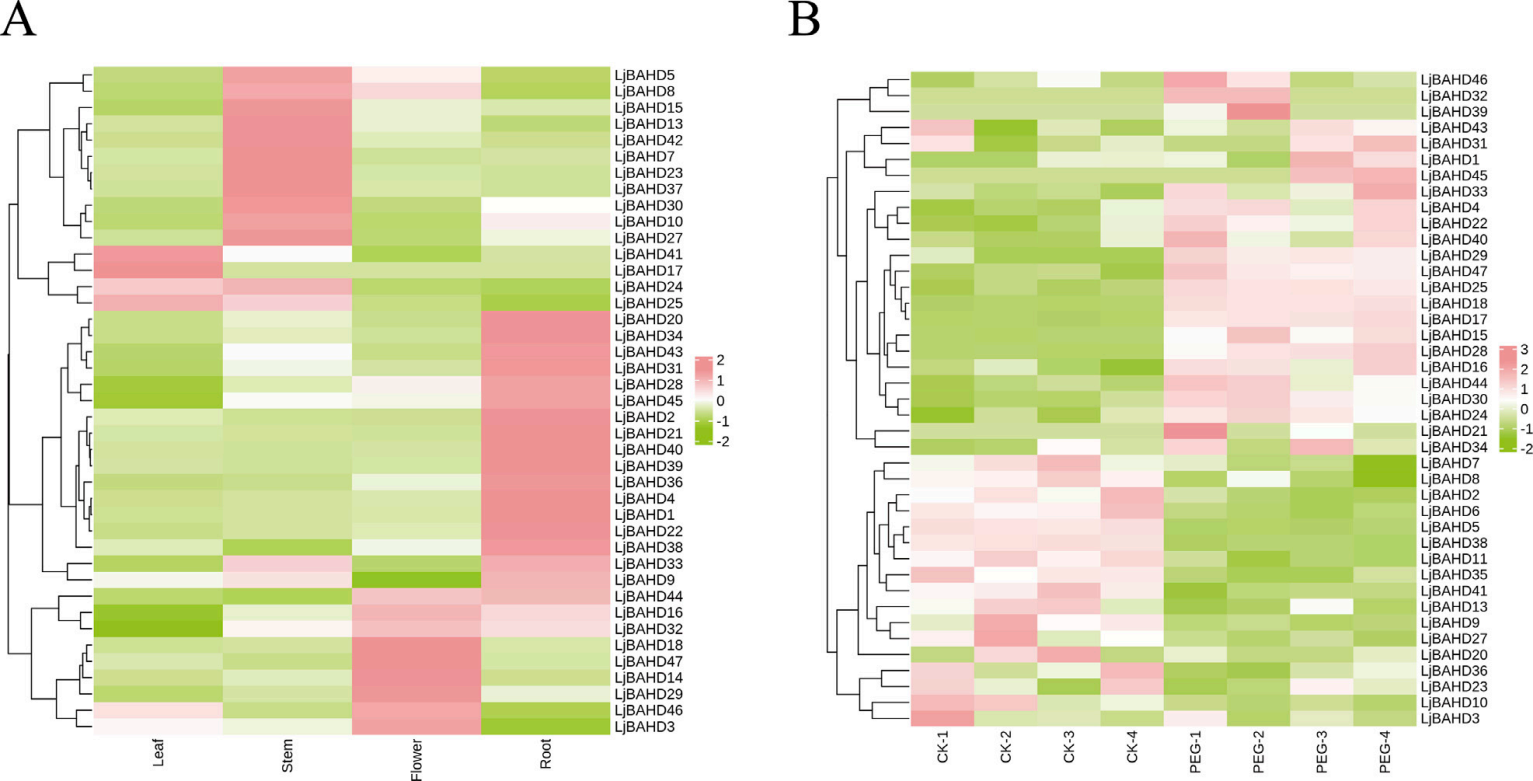
The expression pattern of *LjBAHDs*. **(A)** Expression analysis of the 41 *LjBAHDs* in different tissues of *L. japonicus*. **(B)** Expression pattern of *LjBAHD* genes under drought stress.

Gene expression patterns are intricately linked to the biological functions of genes and can provide insights into their roles in plant growth and adaptation to environmental stress. To ascertain the functional roles of *LjBAHD* genes in *L. japonicus*, an RNA sequencing (RNA-seq) analysis was conducted to examine their organ-specific expression profiles in four key organs: stem, leaf, flower, and root. The expression heatmap for *LjBAHD* genes was generated using the Fragments Per Kilobase of transcript per Million mapped reads (FPKM) values, normalized by row (Figure 6A). The analysis revealed that of the 47 *LjBAHD* genes identified, 41 were transcriptionally active within the examined organs. However, the expression of *LjBAHD6*, *LjBAHD11*, *LjBAHD12*, *LjBAHD19*, *LjBAHD26*, and *LjBAHD35* was not detected. The *LjBAHD* genes exhibited a diverse array of expression patterns. Specifically, three genes were found to be highly expressed in leaves and were classified within Clade I (*LjBAHD17* and *LjBAHD25*) and Clade II (*LjBAHD41*). Twelve genes showed high expression levels in stems, with these genes being distributed across all four Clades. Eight genes were highly expressed in flowers, and these were found across all Clades except Clade IV. Eighteen genes demonstrated high expression in roots, with these genes being distributed across all Clades except Clade I-a, and showing a higher prevalence in Clades I-b and II-b. In order to identify the response of *LjBAHD* family genes to stress treatment, *Leonurus japonicus* plants were subjected to drought stress treatment (Figure 6B). Transcriptome data of leaves showed that the expression of 17 *LjBAHD* genes decreased and the expression of 24 *LjBAHD* genes increased after drought stress treatment. These findings offer a detailed view of the organs-specific expression patterns of *LjBAHD* genes in *L. japonicus*, highlighting their potential roles in various aspects of plant biology and suggesting a complex regulatory network that may be responsive to developmental cues and environmental stimuli.

### 3.7 Characterization and functional analysis of HCT genes in *L. japonicus*

**FIGURE 7:**
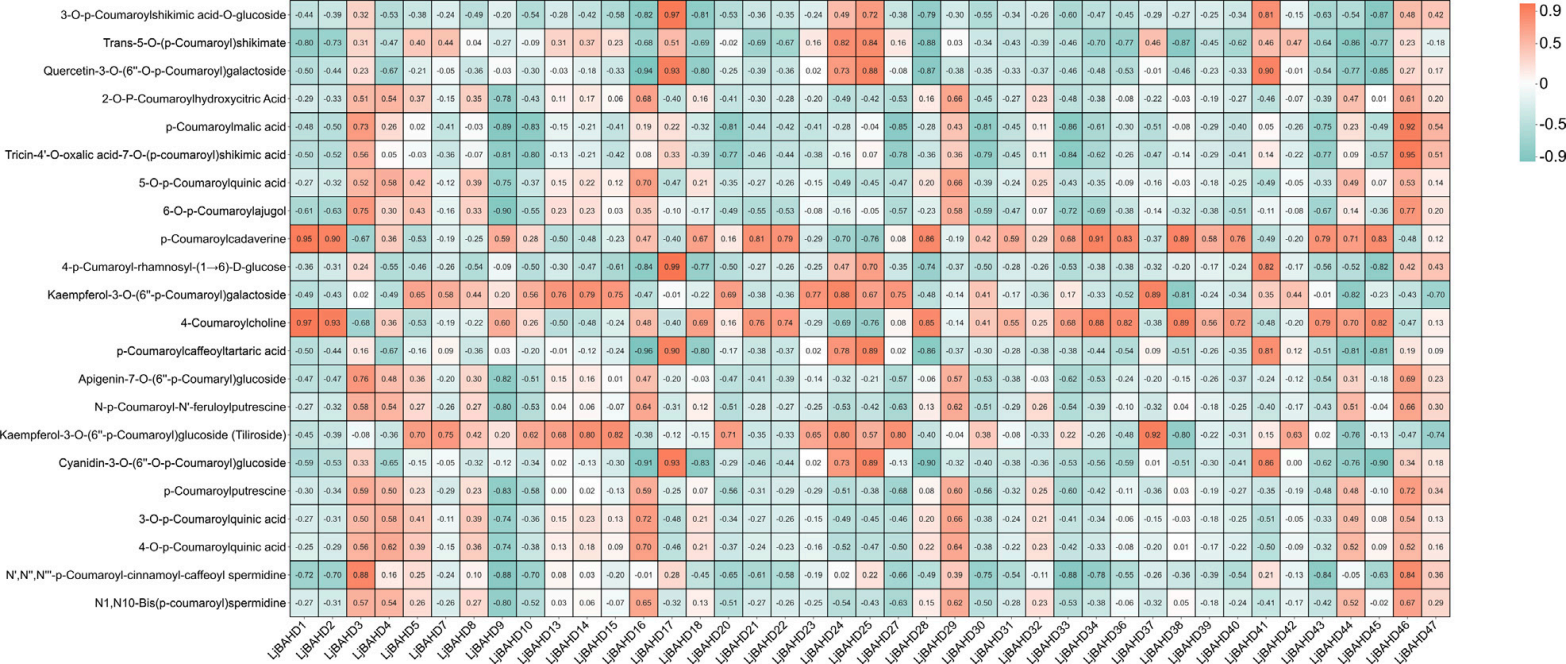
Gene-phenotype data association analysis of 41 *LjBAHDs* and 22 p-coumaroyl analogues.

The enzymatic activities of HCTs are integral to various biosynthetic pathways in plants. In *L. japonicus*, five HCT genes were identified and designated as *LjBAHD4*, *LjBAHD23*, *LjBAHD24*, *LjBAHD25*, and *LjBAHD36* (Supplementary Figure S4). Expression analysis revealed that *LjBAHD4* and *LjBAHD36* were predominantly expressed in roots, while *LjBAHD23*, *LjBAHD24*, and *LjBAHD25* exhibited higher expression levels in the aerial parts of the plant. To elucidate the roles of these HCT genes in the biosynthesis of diverse compounds in *L. japonicus*, a gene-phenotype association analysis was performed. This analysis involved correlating the expression of five HCT and other BAHD genes with the levels of 22 p-coumaroyl analogues detected in the metabolome of *L. japonicus* (Figure 7). The 22 metabolites identified were categorized into four major groups: phenols, flavonoids, terpenoids, and alkaloids, which are known to be the most prevalent classes of compounds in *L. japonicus*. Among the HCT genes, *LjBAHD25* demonstrated the highest expression in leaves and the lowest in roots. Notably, its expression was significantly and positively correlated with 10 p-coumaroyl compounds, suggesting a potential role in the biosynthesis of a wide array of compounds in the aerial parts of *L. japonicus*.

**FIGURE 8:**
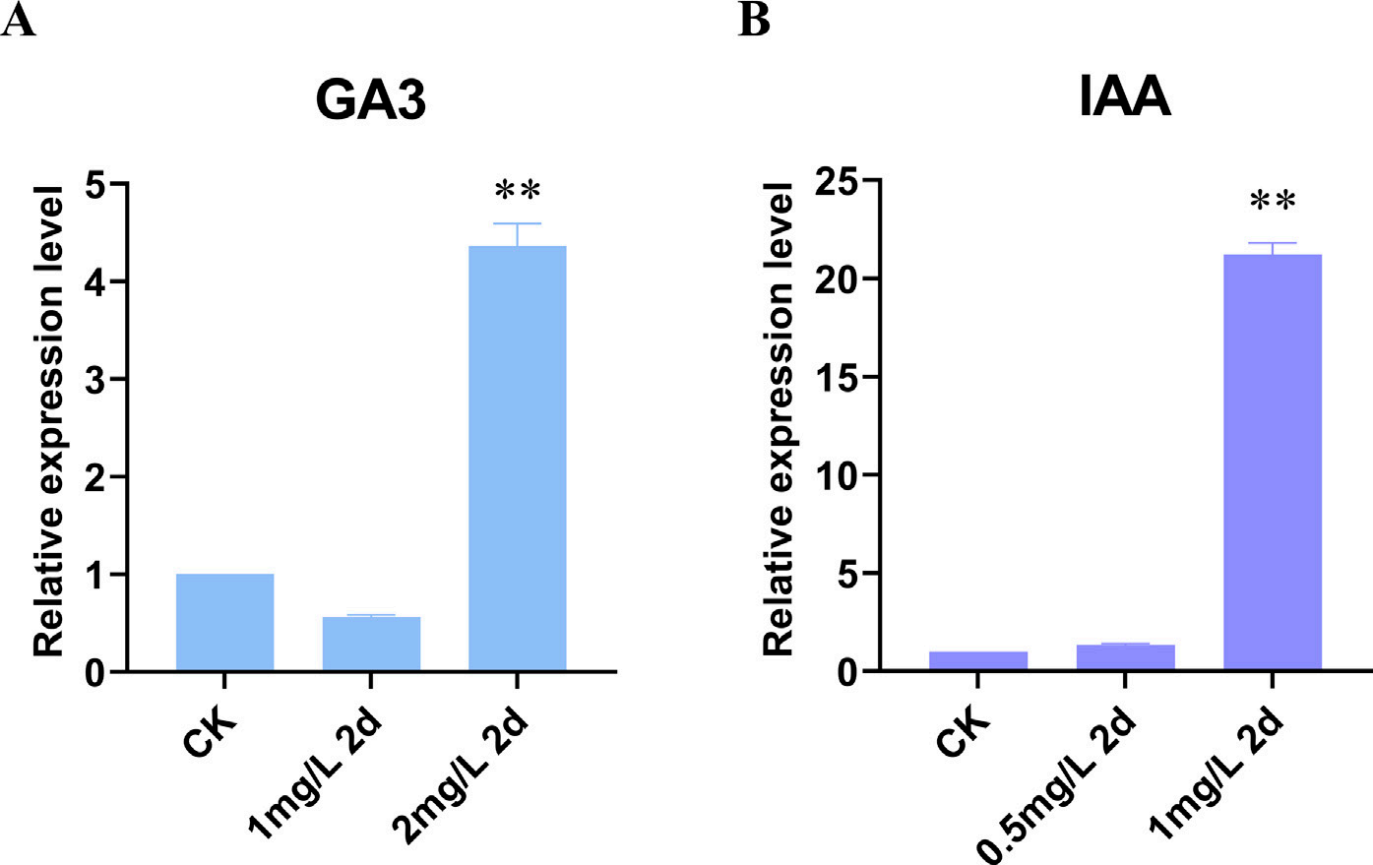
Expression analysis of the *LjBAHD25* in *L. japonicus* under plant hormone.

To investigate the regulation of *LjBAHD25* expression by external factors, a promoter analysis was conducted. This analysis identified two gibberellin-responsive elements and three growth hormone-responsive elements upstream of the *LjBAHD25* gene. Based on these findings, we hypothesized that *LjBAHD25* expression is regulated by gibberellin and growth hormones. The expression levels of *LjBAHD25* were quantified using reverse transcription quantitative polymerase chain reaction (qRT-PCR) following the external application of gibberellin at concentrations of 1 mg/L and 2 mg/L (Figure 8A), and growth hormone at concentrations of 0.5 mg/L and 1 mg/L (Figure 8B). Leaves sprayed with water served as controls. Relative quantification results indicated that *LjBAHD25* expression peaked at a concentration of 2 mg/L gibberellin after 2 days of treatment, which was fourfold higher than that of the control. Upon external application of growth hormone, *LjBAHD25* expression was highest at a concentration of 1 mg/L after 2 days, reaching 21 times the level of the control. These results suggest that *LjBAHD25* expression is hormone-inducible, with growth factors exerting a more pronounced effect on its upregulation.

**FIGURE 9:**
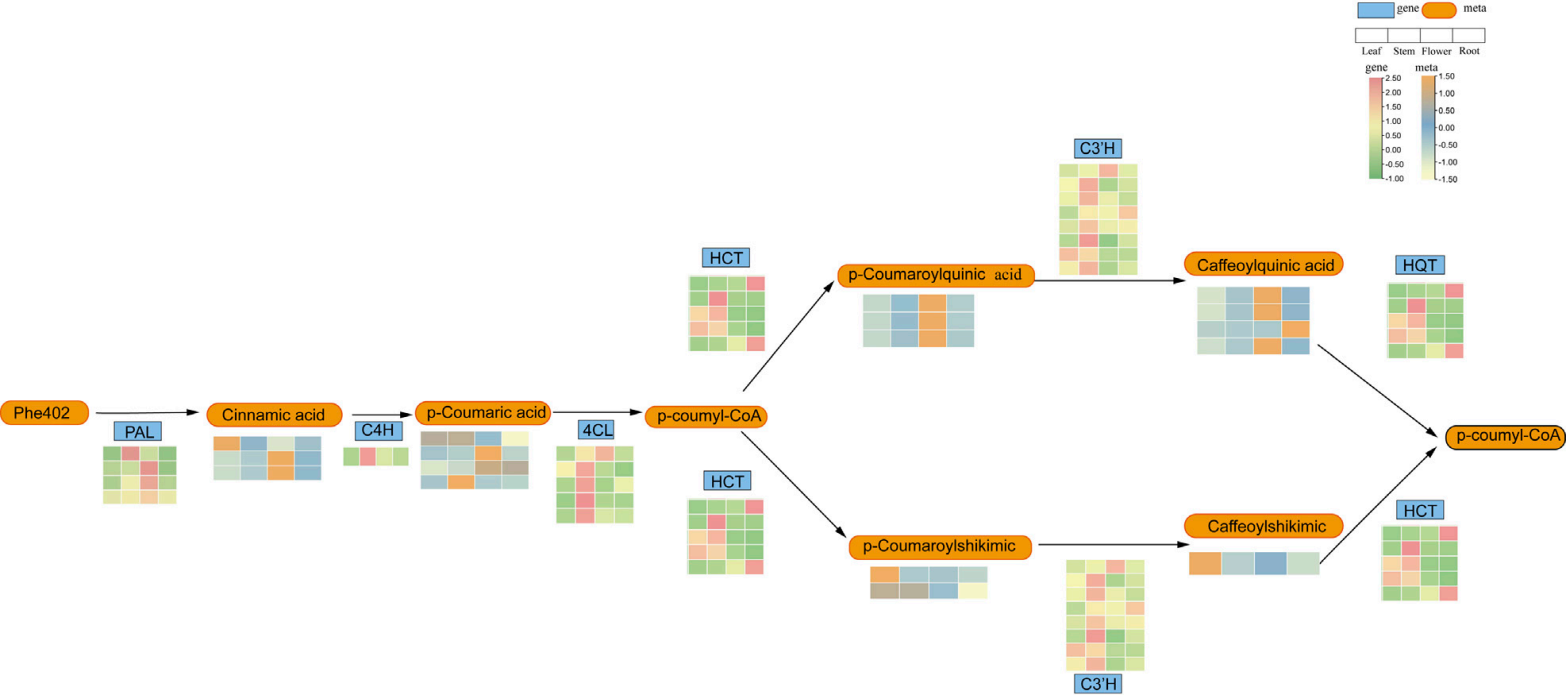
Phenylpropanamine metabolic synthesis pathway in *L. japonicus*.

P-coumaroyl analogues are a subset of phenylpropanoid analogues, which are both plant secondary metabolites synthesised via the phenylpropanoid pathway. To further understand the role of HCT in the phenylpropanoid synthesis pathway in *L. japonicus*, We constructed a core phenylpropanoid metabolism-synthesis pathway combined part of metabolites in Figure 7 and part metabolites (Supplementary Table S4) in core phenylpropanoid pathway and genes in *L. japonicus* (Figure 9). The genes involved in this pathway showed higher expression levels in the aerial parts compared to the roots, and the metabolite content in the aerial parts was also found to be higher than in the root tissues, aligning with the gene expression data. Our findings revealed that the *LjBAHD25* may participate in the core biosynthesis of phenylpropanoid pathway.

## 4 Discussion

*Leonurus japonicus*, an important herb within the repertoire of traditional Chinese medicine, boasts a long-standing history of usage. The bioactive constituents of *L. japonicus* are particularly noteworthy for their therapeutic potential (Miao et al., 2019). Current research underscores the importance of acylation modification in both the primary and secondary metabolic pathways of plants (Moglia et al., 2016). Among the key players in this process is the BAHD acyltransferase family, which is known for its crucial role in these metabolic reactions (Moghe et al., 2023). In this study, we identified 47 BAHD genes in the genome of *L. japonicus* (Figure 1). The genome of *L. japonicus*, with a size of 472.43 Mb, is modestly larger than those of the banana (430 Mb) (Wang et al., 2019), rice (430 Mb), (Yu et al., 2008), and poplar (410 Mb) (Tuskan et al., 2006). Interestingly, despite its larger genome size, only 47 BAHD acyltransferase genes were found in *L. japonicus*, while 46 were found in the banana (Xu et al., 2021), 55 in rice, and 74 in poplar (Tuominen et al., 2011). Gene duplication and amplification contribute to the expansion of gene families. According to the results of gene duplication analyses, 4 genes have syntenic regions in *L. japonicus* (Figure 3A). However, there were 64 BAHDs in *Wurfbainia villosa*, with 19 pairs of genes with syntenic regions between them (Liang et al., 2022). There are 166 BAHD genes in lavender with 32 paired genes, (Zhang et al., 2024), and 123 BAHD genes in *Taxus mairei* with only one paired genes (Xu et al., 2024). This shows that there are diverse evolutionary relationships of BAHD among different species.

BAHD family has a variety of biological functions. These functions include the synthesis of a myriad of secondary metabolites, such as flavonoids, phenols, alkaloids, and anthocyanins, which are essential for the physiology and pharmacological properties of the plant (D’Auria, 2006). The *LjBAHD* genes have been classified into five distinct clades based on their phylogenetic and developmental characteristics, as depicted in Figure 2A. Consistent with the classification in *Welsh Onion*, *Lavandula angustifolia*, and *Lithospermum erythrorhizon*, (Wang et al., 2022; Liu et al., 2023; Zhang et al., 2024), the enzymes are divided into four families in *Theobroma cacao* and six families in *Wurfbainia villosa* and *Taxus mairei* (Abdullah et al., 2021a; Liang et al., 2022; Xu et al., 2024). Each subgroup contains enzymes with similar or different functions. Previous studies have also shown that phylogenetic clustering of the same group does not imply the same function (Nawaz et al., 2019; Abdullah et al., 2021b). It is noteworthy that Clade V, which is predominantly involved in the biosynthesis of volatile esters and includes enzymes capable of synthesizing benzyl benzoate, is absent represented in *L. japonicus* (Figure 2). Thus the absence of this subfamily may underlie the reduced capacity for volatile substance synthesis in *L. japonicus*. This biochemical characteristic correlates with the subtle aroma of the leaves, which is consistent with the descriptions found in traditional Chinese medicinal texts (Wang et al., 2024).

Analysis of the gene structure within the *LjBAHD* family of *L. japonicus* revealed a variable number of introns, ranging from zero to four, with the 74% of *LjBAHD* genes either containing a single intron or being intronless (Supplementary Figure S1). This intron distribution is similar to previous observations across diverse species (Wang X. et al., 2022; Liu et al., 2023; Zhang et al., 2024). Notably, 9 *LjBAHD* genes were identified to contain the motif YFGNC (Supplementary Table S1), which is implicated in the biosynthesis of anthocyanins (Nakayama et al., 2003). Furthermore, the BAHD gene cluster on chromosome 7 was characterized by the presence of motif 7, that is absent in other BAHDs (Figures 1B, 2B). This motif exhibits an exceptionally conserved sequence comprising 50 amino acids. The genes within this cluster are classified under Clade III in the phylogenetic analysis and are closely associated with proteins that possess acetyltransferase activity (Figure 2A). Furthermore, the prediction of subcellular localization showed that LjBAHDs mainly localized in the cytoplasm (Supplementary Table S1), similar to studies in *Lithospermum erythrorhizon* (Wang et al., 2022). This may imply that these proteins perform similar or synergistic functions in the cytoplasm, such as involvement in metabolic pathways, signaling, or protein synthesis. These processes are important in the regulation of *L. japonicus*. In contrast, there are multiple subcellular localizations in *Theobroma cacao* and *Taxus mairei*, including the cytoplasm, chloroplast, and mitochondria (Abdullah et al., 2021a; Xu et al., 2024). This implies that BAHD acyltransferases have different functions in different cellular compartments. Previous studies have also reported that BAHD is involved in pathways related to the regulation of the synthesis of secondary metabolites, such as anthocyanins, lignins, and plant-specific metabolites like paclitaxel (Luo et al., 2007; Li et al., 2018; Kusano et al., 2019). Additionally, BAHD plays a role in the regulation of important plant developmental pathways (Grienenberger et al., 2009).

Studies of the upstream regulatory sequences of *LjBAHDs* (Supplementary Figure S2) revealed the presence of *cis*-acting elements associated with responses to light, phytohormones, various stresses (e.g., low temperature, osmotic stress, and drought), as well as plant growth and development. These findings are consistent with observations from other studies (Liu et al., 2023; Xu et al., 2024). Regulatory elements associated with light and hormone responses are frequently observed in the promoter region of the *LjBAHD* genes (Supplementary Figure S2), suggesting that its expression is tightly regulated by these factors. This regulation is crucial for modulating plant structure and function, as well as the biosynthesis of secondary metabolites (St-Pierre and Luca, 2000). Notably, plant hormones are known to significantly influence the accumulation of secondary metabolites such as alkaloids and phenolics (Zhang et al., 2020). As previous studies have demonstrated, hormone treatments can enhance the accumulation of these compounds in plants. For example, treatment with methyl jasmonate and salicylic acid has been shown to increase the activity of 10-deacetylbaccatin III-10-O-acetyltransferase, thereby enhancing paclitaxel content in *Picea rubra* (Zhang et al., 2010). Similarly, jasmonic acid has been reported to stimulate paclitaxel synthesis in *Picea rubra* cells (Baebler et al., 2002). Additionally, methyl jasmonate treatment has induced the expression of *LaBAHDs*, leading to an increased accumulation of linalyl acetate and lavandin acetate in *Lavandula angustifolia* (Zhang et al., 2024). In this study, *LjBAHD25* was categorized as a HCT enzyme, a class of enzymes that have been extensively implicated in the biosynthesis of phenylpropanoid (Figure 9). This pathway is a critical conduit to produce a variety of plant metabolites. The downstream products of this biosynthetic route encompass several key compounds, including anthocyanins, chlorogenic acid, and lignin (Besseau et al., 2007; Xing et al., 2017; Zheng et al., 2023). A detailed analysis of the promoter region of the *LjBAHD25* gene revealed an abundance of light-responsive and hormone-responsive elements (Supplementary Figure S2). Moreover, *LjBAHD25* exhibited a positive response to auxins and gibberellins hormone treatments (Figure 8), which is essentially consistent with the role of BAHD acyltransferases in secondary metabolism. These findings suggest that the *LjBAHD25* gene may modulate its expression levels in response to shifts in the external environment, thereby regulating the metabolic processes of *L. japonicus* and enhancing its adaptability to environmental fluctuations. Of course, further experiments are needed to determine which secondary metabolite biosynthesis in *L. japonicus* is associated with this gene and whether this association is directly consistent with the presence of *cis*-acting elements. Plant responses to biotic and abiotic stresses involve a highly complex network of gene regulation, a process often mediated by multiple transcription factors. The accumulation of hydroxycinnamoyl derivatives, usually esters, in many plant species is thought to defend against both types of stress (Sullivan, 2017). The presence of transcription factor binding sites associated with MYB, MYC, and WRKY in LjBAHDs (Figure 4C) further suggests that transcription factor-based signal transduction networks play a critical role in these processes.

We found that drought stress significantly affected the expression of BAHD genes (Figure 6B). Similarly, a drought stress response in BAHD genes was observed in legume alfalfa (Yu et al., 2008). Drought and salinity stresses induced the expression of *LaBAHDs*, leading to an increased accumulation of linalyl acetate and lavender acetate in lavender (Zhang et al., 2024). Additionally, Xu et al. reported that *MaBAHD* genes participate in responses to multiple abiotic stresses, such as cold, salt, and osmotic treatments in banana (Xu et al., 2021). Previous studies have also highlighted the role of BAHD acyltransferases in responding to microbial pathogens and herbivorous pests (Zheng et al., 2009; Qiao et al., 2024). Thus, these findings further support the critical role of the BAHD family in plant adaptation to terrestrial environments, response to abiotic stresses, and interactions with biotic factors.

Considering these results, future cultivation strategies for *L. japonicus* could potentially involve the topical application of growth factors or gibberellins to bolster the levels of secondary metabolites. Such an approach could have important implications for the enhancement of medicinal value and overall productivity of *L. japonicus*.

## 5 Conclusion

In this study, we identified 47 BAHD genes in *L. japonicus* and analyzed the gene location, gene structure, conserved motif, phylogenetic relationship, promoter elements and gene expression using bioinformatics methods. Our findings revealed that the members of the BAHD gene family, may play crucial roles in the biosynthesis of phenylpropanoid pathway and in abiotic stress response. Generally, studying the molecular characteristics of BAHD gene family contributes to the understanding of their secondary metabolite synthesis function in *L. japonicus*, which provided a theoretical basis for breeding *L. japonicus* with high medicinal value.

## Data Availability

The datasets presented in this study can be found in online repositories. The names of the repository/repositories and accession number(s) can be found in the article/Supplementary Material.
