# Supplementary material for: Genome-wide identification and expression analysis of the BAHD gene family in Leonurus japonicus
Source: Front Genet. 2024 Dec 19;15:1512692. doi: 10.3389/fgene.2024.1512692 (PMC11693612; doi:10.3389/fgene.2024.1512692)
Supplement: Supplementary file 1 [file Table1.doc]

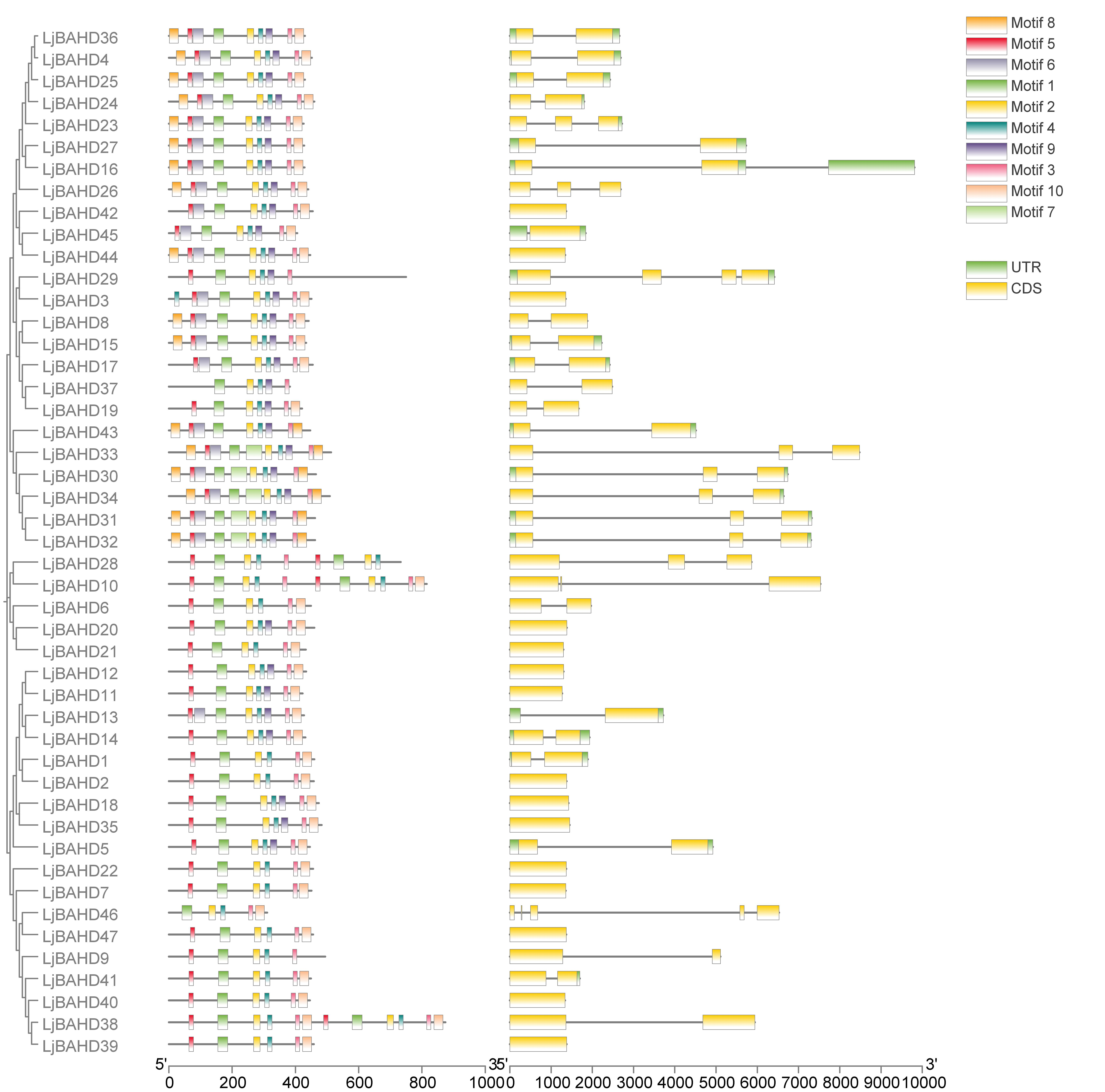


Figure S1 Gene structural analyses of the *LjBAHDs.*

*
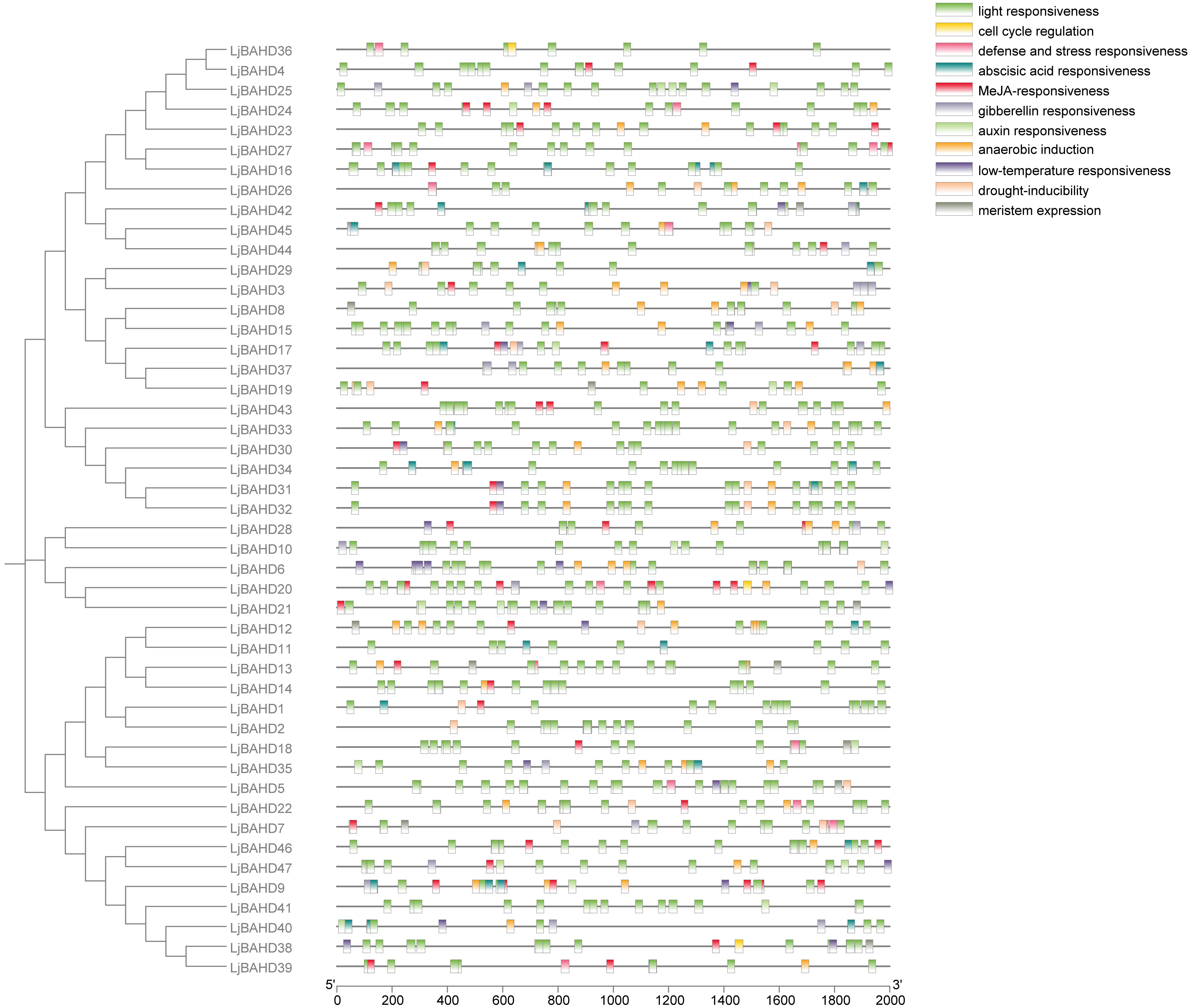
*

Figure S2 Distribution of *cis*-elements in 47 *LjBAHDs* promoter regions.


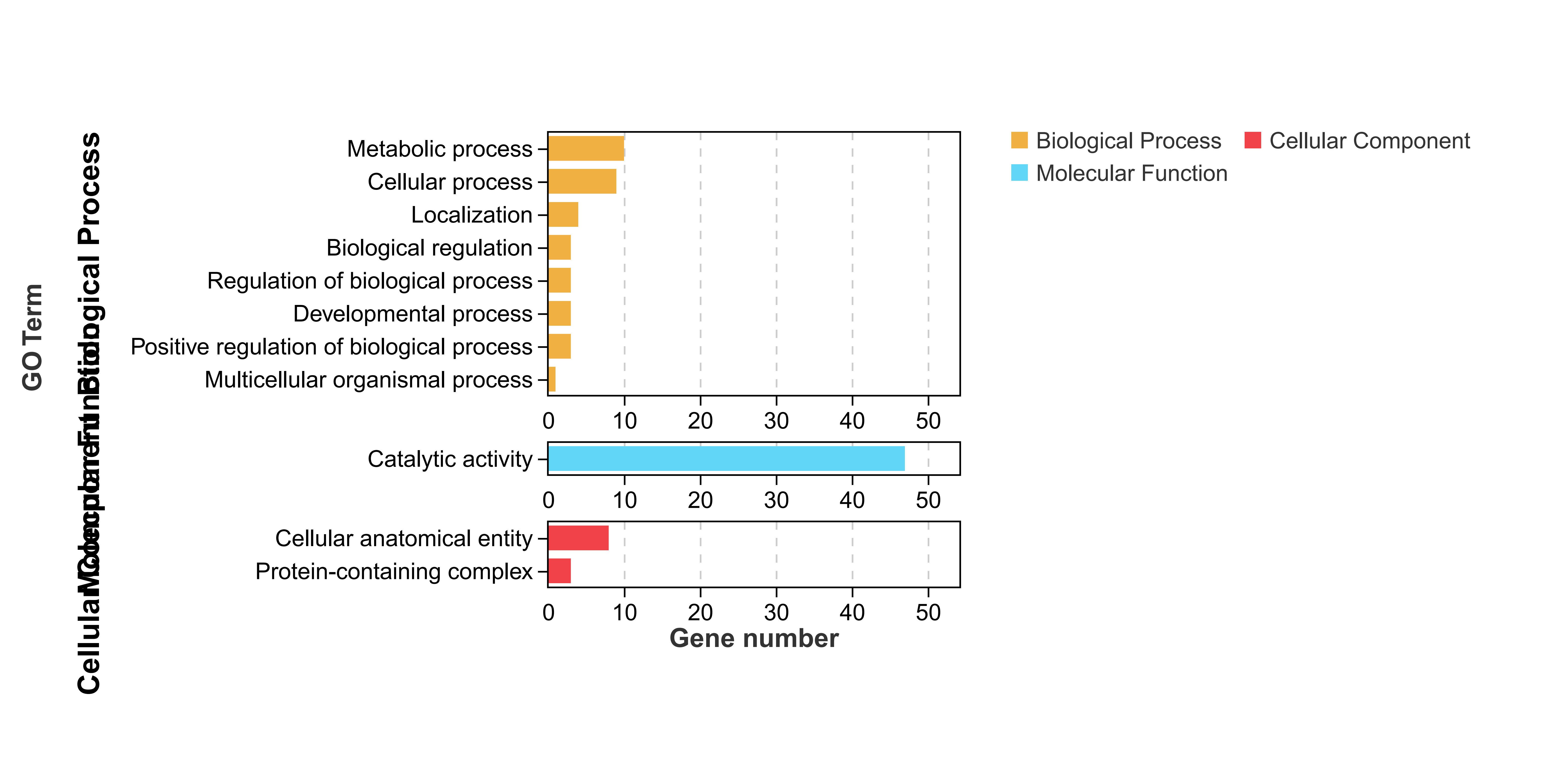


Figure S3 Functional enrichment analysis of 47 *LjBAHDs* in *L. japonicus* by gene ontology (GO).


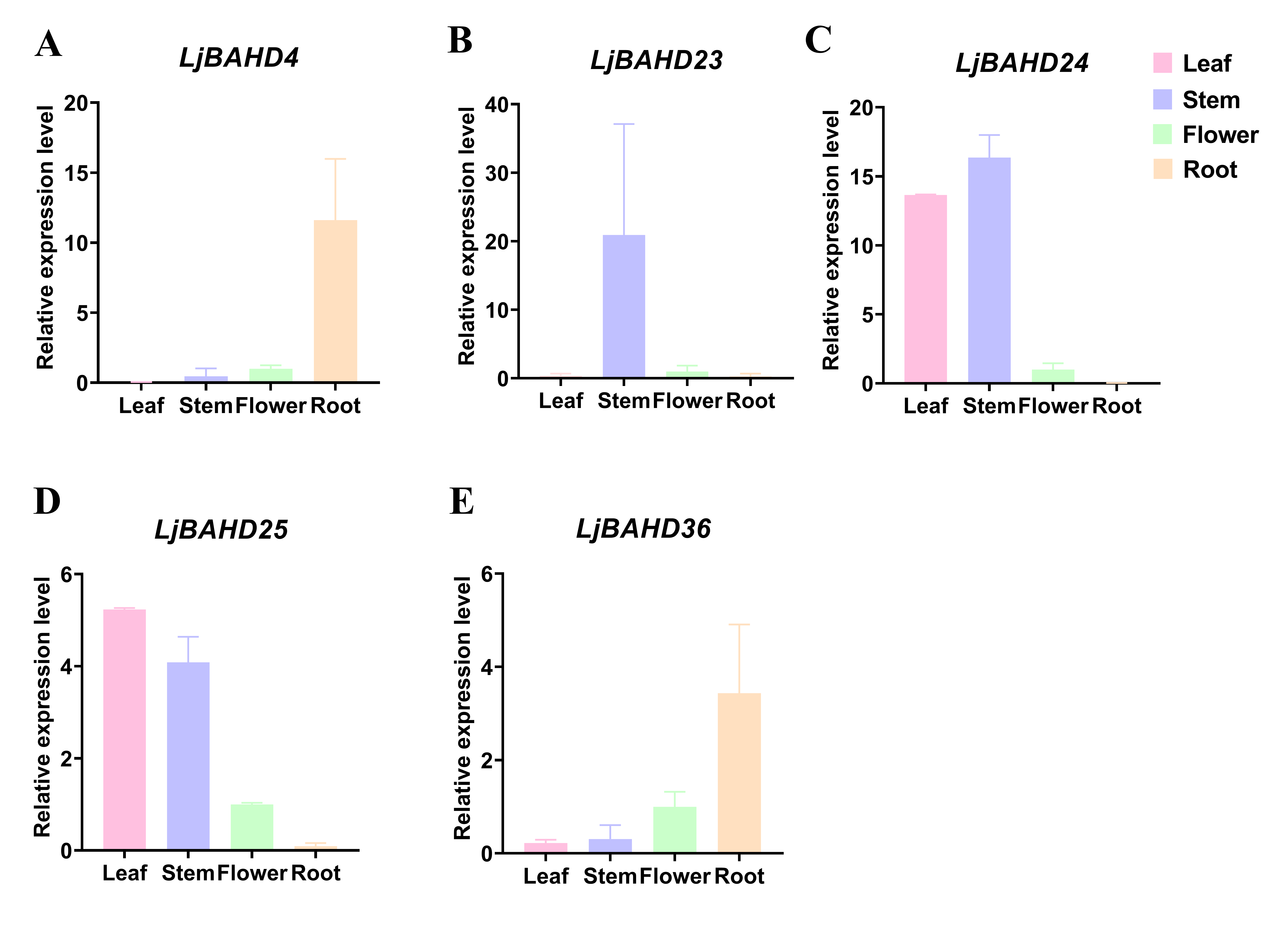


Figure S4 Expression analysis of the 5 HCTs in different tissues of *L. japonicus.*

Table S1 Detailed information of all identified LjBAHDs

| **Genome number** | **Gene name** | **Chromosomal Location** | **Gene starting site** | **Gene end site** | **Number of amino acids** | **Molecular weight/Da** | **pI** | **Subcellular location prediction** | **YFGNC motif** |
| --- | --- | --- | --- | --- | --- | --- | --- | --- | --- |
|
| LjH_00326-R1 | LjBAHD1 | Chr1 | 3356693 | 3358584 | 460 | 50.9 | 5.27 | Cyto |  |
| LjH_00329-R1 | LjBAHD2 | Chr1 | 3393765 | 3395141 | 458 | 51.1 | 6.1 | Cyto |  |
| LjH_00921-R1 | LjBAHD3 | Chr1 | 33450538 | 33451893 | 451 | 50.7 | 5.72 | Cyto |  |
| LjH_01126-R1 | LjBAHD4 | Chr1 | 33450538 | 33451893 | 452 | 50.3 | 6.22 | Cyto |  |
| LjH_01709-R1 | LjBAHD5 | Chr1 | 44725169 | 44730089 | 446 | 49.2 | 5.2 | Cyto |  |
| LjH_02129-R1 | LjBAHD6 | Chr1 | 48209897 | 48211866 | 449 | 50.2 | 5.17 | Cyto |  |
| LjH_02201-R1 | LjBAHD7 | Chr1 | 48866855 | 48868210 | 451 | 50.4 | 8.15 | Cyto | √ |
| LjH_02514-R1 | LjBAHD8 | Chr1 | 51405991 | 51407875 | 442 | 49 | 5.91 | Cyto |  |
| LjH_03304-R1 | LjBAHD11 | Chr3 | 29095335 | 29096606 | 423 | 46.9 | 6.19 | Cyto |  |
| LjH_03310-R1 | LjBAHD12 | Chr3 | 29270448 | 29271752 | 434 | 47.4 | 6.09 | Cyto |  |
| LjH_03324-R1 | LjBAHD13 | Chr3 | 29943937 | 29947659 | 427 | 47.8 | 5.91 | Cyto |  |
| LjH_03325-R1 | LjBAHD14 | Chr3 | 29957416 | 29959349 | 432 | 48.3 | 5.91 | Cyto |  |
| LjH_03626-R1 | LjBAHD15 | Chr3 | 34800285 | 34802514 | 434 | 48.4 | 5.27 | Cyto |  |
| LjH_03750-R1 | LjBAHD16 | Chr3 | 35973574 | 35983386 | 429 | 48 | 7.63 | Cyto |  |
| LjH_05960-R1 | LjBAHD18 | Chr5 | 2875090 | 2876514 | 474 | 52 | 8.41 | Cyto |  |
| LjH_06398-R1 | LjBAHD19 | Chr5 | 22922716 | 22924389 | 421 | 47.2 | 6.76 | Cyto |  |
| LjH_06804-R1 | LjBAHD20 | Chr5 | 37970812 | 37972194 | 460 | 51.5 | 5.79 | Cyto |  |
| LjH_06809-R1 | LjBAHD21 | Chr5 | 38007047 | 38008348 | 433 | 47.6 | 5.19 | Cyto |  |
| LjH_11210-R2 | LjBAHD17 | Chr4 | 34233586 | 34236518 | 455 | 51.1 | 6.47 | Cyto | √ |
| LjH_11892-R1 | LjBAHD22 | Chr6 | 1465314 | 1466684 | 456 | 50.4 | 5.5 | Cyto | √ |
| LjH_11976-R3 | LjBAHD23 | Chr6 | 2814967 | 2817689 | 426 | 47.2 | 6.23 | Cyto |  |
| LjH_11977-R1 | LjBAHD24 | Chr6 | 2835224 | 2837027 | 460 | 51.4 | 6.42 | Cyto |  |
| LjH_11978-R1 | LjBAHD25 | Chr6 | 2881699 | 2884129 | 430 | 47.9 | 7.14 | Cyto |  |
| LjH_11979-R1 | LjBAHD26 | Chr6 | 2892058 | 2894747 | 441 | 49.1 | 6.04 | Cyto |  |
| LjH_11982-R1 | LjBAHD27 | Chr6 | 2955234 | 2960964 | 428 | 47.2 | 5.73 | Cyto |  |
| LjH_12283-R1 | LjBAHD28 | Chr6 | 19813185 | 19819049 | 733 | 81.8 | 6.54 | Cyto |  |
| LjH_14222-R1 | LjBAHD9 | Chr2 | 767430 | 772539 | 494 | 55.2 | 5.76 | Cyto | √ |
| LjH_15470-R1 | LjBAHD10 | Chr2 | 10869632 | 10877165 | 815 | 91.4 | 5.91 | Cyto |  |
| LjH_16856-R1 | LjBAHD29 | Chr7 | 976282 | 982696 | 750 | 82.8 | 5.56 | Cyto |  |
| LjH_17339-R1 | LjBAHD30 | Chr7 | 21478708 | 21485449 | 465 | 51.6 | 6.04 | Cyto |  |
| LjH_17352-R1 | LjBAHD31 | Chr7 | 22255188 | 22262512 | 462 | 51.4 | 5.69 | Cyto |  |
| LjH_17354-R1 | LjBAHD32 | Chr7 | 22321074 | 22328380 | 462 | 51.3 | 5.69 | Cyto |  |
| LjH_17377-R2 | LjBAHD33 | Chr7 | 23512985 | 23521552 | 513 | 57.6 | 6.96 | memb |  |
| LjH_17381-R1 | LjBAHD34 | Chr7 | 23916494 | 23923133 | 509 | 56.8 | 7.99 | Cyto |  |
| LjH_20785-R1 | LjBAHD35 | Chr8 | 36800895 | 36802346 | 483 | 53.3 | 8.25 | Cyto |  |
| LjH_21538-R1 | LjBAHD36 | Chr9 | 22891867 | 22894525 | 430 | 47.9 | 6.56 | Cyto |  |
| LjH_21676-R1 | LjBAHD37 | Chr9 | 26554411 | 26556892 | 383 | 42.7 | 8.32 | Cyto |  |
| LjH_21955-R1 | LjBAHD38 | Chr9 | 29740124 | 29746067 | 874 | 96.6 | 5.62 | Cyto | √ |
| LjH_21956-R1 | LjBAHD39 | Chr9 | 29751146 | 29752522 | 458 | 50.4 | 5.85 | Cyto | √ |
| LjH_21957-R1 | LjBAHD40 | Chr9 | 29753947 | 29755287 | 446 | 49.1 | 6.18 | Cyto | √ |
| LjH_21970-R1 | LjBAHD41 | Chr9 | 29873986 | 29875685 | 449 | 49.6 | 6.21 | Cyto | √ |
| LjH_22071-R2 | LjBAHD42 | Chr9 | 30819328 | 30820695 | 455 | 50.9 | 6.72 | Cyto |  |
| LjH_23895-R1 | LjBAHD43 | Chr10 | 24915960 | 24920474 | 447 | 49.5 | 6.28 | Cyto |  |
| LjH_24092-R1 | LjBAHD44 | Chr10 | 27078382 | 27079725 | 447 | 49.4 | 6.52 | Cyto |  |
| LjH_24093-R1 | LjBAHD45 | Chr10 | 27089774 | 27091614 | 406 | 44.6 | 6.34 | Cyto |  |
| LjH_25129-R1 | LjBAHD46 | Chr10 | 35449235 | 35455763 | 311 | 33.8 | 5.14 | Cyto | √ |
| LjH_25130-R3 | LjBAHD47 | Chr10 | 35457583 | 35458953 | 456 | 50.5 | 5.85 | Cyto |  |

Table S2 Segmental replication and tandem repeat replication covariate gene pair Ka/Ks analysis of *LjBAHDs*

| Gene pair name | | Gene pair ID | | Ka | Ks | Ka/Ks | Duplications type |
| --- | --- | --- | --- | --- | --- | --- | --- |
| *LjBAHD6* | *LjBAHD18* | LjH_02129-R1 | LjH_05960-R1 | 0.7834 | 4.5609 | 0.1718 | segmental duplication |
| *LjBAHD9* | *LjBAHD38* | LjH_14222-R1 | LjH_21955-R1 | 0.3568 | 2.0656 | 0.1727 | segmental duplication |

Table S3 Primer pairs used for qRT-PCR analysis on the target sequences in *LjBAHD*

| **Gene name** | **primer** | |
| --- | --- | --- |
|  | Forward primer (5'-3') | Reverse primer (5'-3') |
| *LjBAHD25* | CCCGCCGTCGATTATTCTCA | AAAGAATGGAGACGCGCTGA |
|  | TCGAGAAGACCTCAGCAACTG | TCGAGAAGACCTCAGCAACAC |

Table S4 The detailed names and CAS numbers of the compounds in Figure 9

| Compounds | CAS |
| --- | --- |
| 3-O-p-Coumaroylshikimic acid-O-glucoside | - |
| Trans-5-O-(p-Coumaroyl)shikimate | - |
| 5-O-p-Coumaroylquinic acid* | 1899-30-5 |
| 3-O-p-Coumaroylquinic acid* | 87099-71-6 |
| 4-O-p-Coumaroylquinic acid | 32451-86-8 |
| 5-O-Caffeoylshikimic acid | 180981-12-8 |
| Neochlorogenic acid (5-O-Caffeoylquinic acid) | 906-33-2 |
| Cryptochlorogenic acid (4-O-Caffeoylquinic acid) | 905-99-7 |
| 1-Caffeoylquinic acid | 1241-87-8 |
| Chlorogenic acid (3-O-Caffeoylquinic acid) | 327-97-9 |
| p-Coumaric acid-4-O-glucoside | 117405-48-8 |
| p-Coumaric acid ethyl ester | 7362-39-2 |
| p-Coumaric acid | 501-98-4 |
| p-Coumaric acid methyl ester | 3943-97-3 |
| 3,4-Dimethoxycinnamic acid | 2316-26-9 |
| 2-Hydroxycinnamic acid* | 583-17-5 |
| α-Hydroxycinnamic Acid* | 5801-57-0 |
